# At Least Three Transporters Likely Mediate Threonine Uptake Needed for Mouse Embryonic Stem Cell Proliferation

**DOI:** 10.3389/fcell.2016.00017

**Published:** 2016-03-15

**Authors:** Tara M. Formisano, Lon J. Van Winkle

**Affiliations:** Department of Biochemistry, Midwestern University College of Health ScienceDowners Grove, IL, USA

**Keywords:** mES cells, cell proliferation, threonine, amino acid transport system ASC, amino acid transport system L

## Abstract

Stem cells are at the forefront of current regenerative and biomedical research. Thus, there exists an imperative and urgent need to understand the mechanisms that drive stem cell function in order to exploit their use as a therapeutic tool. Amino acids are potent inducers of signaling cascades that drive stem cell proliferation and differentiation. With a focus on mouse embryonic stem (mES) cells, Threonine (Thr) is the only amino acid required in culture media for mES cell proliferation. Current research associates this need for Thr with threonine dehydrogenase (TDH), which catabolizes Thr to glycine and acetyl-CoA in mES cells. This theory depends, in part, on the ability of 3- hydroxynorvaline (3-HNV) to inhibit both TDH and mES cell proliferation. However, the concentration of 3-HNV needed to inhibit mES cell proliferation is more than an order of magnitude less than its apparent K_i_ for TDH inhibition. Additionally, 3-HNV inhibits human embryonic stem (hES) cell proliferation, but hES cells do not express a functional *tdh* gene. Such findings indicate another mechanism for Thr stimulated mES and hES cell proliferation. Since amino acid transporters may be inducers of signaling cascades, we characterized the Thr transport systems in mES cells. We found that there is a Na^+^-dependent and a Na^+^-independent component of substrate-saturable transport, with the Na^+^-dependent component predominating. We also found that of 20 amino acids tested, the amino acids that were the strongest inhibitors of the Na^+^-dependent component of radiolabeled Thr transport were Ser, Cys, 4-OH-Pro, Asn, Met, and non-radiolabeled Thr itself. Such findings are consistent with characteristics of the ASC transport system, suggesting that this ASC system is responsible for the majority of Thr transport in mES cells. We confirmed expression of mRNA encoding the ASC system transporters, ASCT1 and ASCT2, in mES cells using RT-PCR. In conclusion, mES cells likely express at least three transporters of Thr; at least two Na^+^-dependent transporters and one Na^+^-independent one.

## Introduction

Stem cells are one of the primary focuses in current regenerative medicine and biomedical research. With their vast developmental potential, proliferative competence, and genetic stability, stem cells have noteworthy therapeutic potential. In fact, stem cells have the potential to treat a wide variety of diseases and injuries including neurological disease, blindness, Type I Diabetes Mellitus, Parkinson's disease, Leukemia, and spinal cord injuries (Amit et al., 2000; Puri and Nagy, 2012). Thus, there is a race to understand stem cell biology in order to exploit their potential uses.

In an effort to understand stem cell function, some research has focused on the role of amino acids in stem cell proliferation and differentiation (Van Winkle, 2013). mES cell proliferation and maintenance of the undifferentiated state depends exclusively on the presence of Thr in the culture medium (Alexander et al., 2011). These authors proposed a need for Thr owing to its rapid catabolism via threonine dehydrogenase (TDH), an enzyme abundant in mES cells. The selective TDH inhibitor, 3-HNV, inhibits mES cell proliferation, and Thr rescues the cells from this inhibition (Wang et al., 2009). A sharp contradiction, however, can be seen when comparing mES cells and hES cells. hES cells seem to rely in a similar way on Thr for proliferation, but hES cells produce inactive TDH (Darling et al., 2000; Edgar, 2002; Van Winkle et al., 2014). In addition, the Thr and 3-HNV concentrations needed for mES cell proliferation or inhibition of proliferation, respectively, are more than an order of magnitude less than the apparent K_m_ and K_i_ values for the interaction of Thr or 3-HNV with TDH (Van Winkle et al., 2014). These results support the conclusion that TDH is not the site of 3-HNV and Thr action in hES cells, and it may not be the only site of their action in mES cells.

Previous studies attribute the need for Thr by mES cells to its rapid catabolism via TDH. These studies found that the mRNA levels of TDH were 1000 times higher in mES cells compared to differentiated cells (Ochocki and Simon, 2013). Much research is consistent with the conclusion that TDH catabolism of Thr is responsible for the unique one carbon metabolic state of mES cells (Han et al., 2013). Specifically, TDH is a mitochondrial enzyme that hydrolyzes Thr into glycine and acetyl-coA (Han et al., 2013). Glycine is used to promote mES cell one carbon metabolism while acetyl-coA is oxidized in the TCA cycle (Han et al., 2013). This one carbon metabolism supports the purine and the pyrimidine (thymidine) biosynthesis needed for mES cell proliferation (Ochocki and Simon, 2013). Furthermore, inhibition of TDH eventually causes mES cell death (Han et al., 2013). Thus, Thr breakdown products supply metabolic pathways with substrates needed for rapid mES cell proliferation.

Threonine's role in mES cell proliferation is likely more complex, however. Studies indicate that Thr is a very potent activator of several critical signaling pathways. One such study explored Thr's role in regulating embryonic stem (ES) cell transition through the G1/S phase of the cell cycle (Ryu and Han, 2011). Specifically, this study found that Thr stimulates ES cell transition through the G1/S phase by activating lipid raft/caveolae-dependent signaling pathways, including PI3K/Akt, MAPKs, mTOR, p7056k, and 4E-BP1 (Ryu and Han, 2011). The induction of these signaling pathways leads to the transcription of c-Myc, which is a potent regulator of cell proliferation (Ryu and Han, 2011). Moreover, this study also found that Thr will restore and increase cyclin D1 and cyclin E, both of which are the rate limiting activators on the G1 to S phase transition in the cell cycle required for proliferation (Ryu and Han, 2011). The lipid raft dependent signaling could be through a direct association of Thr and its transporter with the raft (Ryu and Han, 2011). Thr also may be needed intracellularly for signaling, which would depend on uptake of Thr by the cells (Ryu and Han, 2011). All of these studies support the need to characterize Thr transport in ES cells.

Amino transport systems were originally categorized according to their substrate selectivity (cationic, anionic, or zwitterionic amino acids) and Na^+^ dependence (Van Winkle, 2013). Numerous amino acid transport systems have been characterized and the transporter proteins comprising the systems identified (Placín et al., 1998; Van Winkle, 2013). Systems that could transport Thr well include the Na^+^–independent systems, L (named for Leu) and b^0, +^ (selective for Arg), and the Na^+^-dependent systems, B^0, +^ (selective for Leu, Ile, and Trp), A (named for Ala) and ASC (named for Ala, Ser, and Cys; Van Winkle, 2013). Of these, system ASC is particularly attractive as a possible Thr transporter in mES cells because this system tolerates hydroxyl and sulfahydryl groups at C3 or C4 very well (Utsunomiya-Tate et al., 1996; Pinilla-Tenas et al., 2003). Thr has such a hydroxyl group rendering if a good substrate for this system. Nevertheless, when amino acid transport has not been studied in a particular cell type, it should be characterized first, rather than assuming that certain amino acid transporters are present in those cells (Van Winkle, 2013). Since Thr transport has not been characterized previously in mES cells, we began to characterize it as described in these studies.

## Methods

### mES cell culture

ATCC American Tissue Type Culture Collection CE1 Mouse Embryonic Stem Cells were placed in T75 Tissue Culture Flasks with mES cell media for 2 days. Media included DMEM F12 (Gibco), Leukemia Inhibiting Factor (Gibco), Fetal Bovine Serum (Bio west), non-essential amino acid (Gibco), Penicillin-streptomycin (Hydroclone) and glutamine solution containing DMEM F12 (Gibco), glutamine (Sigma), and 2-mercaptoethanol (Bio Rad). After 2 days of growth, the cells were trypsinized with Tryspin-EDTA (Gibco) for 5 min and then collected in a 15 ml conical centrifuge tube. The cells were spun down at 3400 rpm for 1 min and then re-suspended in 5 ml of mES cell media by trituration. Cells were then placed in approximately 20 ml of mES cell media in a 50 ml conical tube and counted using a TC20 Automated Cell Counter (Bio Rad). They were then brought to a concentration of 2 × 10^5^ cells/ml mES cell media. 1 ml of mES cell suspension was plated into each well in a 6 well tissue culture treated plate and then placed in 5% CO_2_, 37°C, 100% humidity incubator (Binder) for 1 day. On day 2, media was replaced with fresh mES cell media. The cells were placed back into the incubator. On day 3, experimental procedures were performed.

### Time course

mES cell media was aspirated off of the 6 well plates containing mES cells using an in house vacuum system. 1 ml of the different uptake buffers (with Na^+^ and without Na^+^) containing 50 uM-radioactive labeled Thr was added to their respective wells. The uptake buffers tested include those containing 140 mM NaCl, LiCl, Choline Cl, and KCl, and 280 mM Mannitol. The uptake buffer stock solution also contained 25 mM HEPES+ Tris (pH = 6.8), 5.4 mM KCl, 0.8 mM MgSO_4_, 5 mM glucose, 1.8 mM CaCl_2_, and 50 uM [^3^H]-Thr (20 Ci/mmol in stock solution supplied by American Radiolabeled Chemicals, Saint Louis, MO). The cells were incubated up to 10 min at 37°C, 100% Humidity (Napco). After each incubation period, 20 uL of the sample was taken from each well and placed into scintillation vials. The remaining radioactive treatment was aspirated off. Cells were then washed 6 times with the appropriate with Na^+^ or without Na^+^ uptake buffer (NOTE: This buffer did not contain any radiolabeled Thr). The 6th wash was collected and placed in a second set of scintillation vials to check for thoroughness of washing. The cells were then treated with 1 ml 4% SDS detergent for 2 min. The sample was collected into a 3rd set of scintillation vials. 5 ml of Scintillation cocktail Econ2 (Fischer Scientific) was added to all scintillation vials. All vials were placed in a Beckmann LS6500 Beta liquid scintillation counter and counted for 10 min. Experiments were performed 1–3 times with 1–3 replicate determinations as indicated in Figure legends.

### Radiolabeling amino acid inhibitor experiment

mES cell media was aspirated off of the 6 well plates containing mES cells using an in house vacuum system. A radioactive solution containing uptake buffer, 50 uM radiolabeled Thr, and 10 mM of various potential amino acid inhibitors (Glu, Ser, Lys, Gly, Leu, MeAIB, Pro, Asp, Thr, Sar, Cys, Asn, Ala, Met, His, Gln, Arg, Tyr, BCH at 5mM, 4-OH-Pro) was prepared. Each study was repeated using the Na^+^ containing uptake buffer and with the no-Na^+^ containing uptake buffer, Mannitol. The stock uptake buffer solution contained 25 mM HEPES + Tris (pH = 6.8), 5.4 mM KCl, 0.8 mM MgSO_4_, 5 mM glucose, 1.8 mM CaCl_2_, and 50 uM [^3^H]-Thr. The Na^+^ containing buffer also contained 140 mM NaCl and the no-Na^+^ containing uptake buffer also contained 280 mM Mannitol. 1 ml of the radiolabeled Thr uptake solution was added to the appropriate well in the 6 well plates and then incubated for 10 min at 37°C, 100% humidity. After 10 min, 20 uL of the sample was taken from each well and placed into scintillation vials. The remaining radioactive treatment was aspirated off. The cells were washed 6 times with the appropriate NaCl or Mannitol uptake buffer. The 6th wash was collected and placed in a second set of scintillation vials to check for thoroughness of washing. The cells were then treated with 1 ml 4% SDS detergent for 1 min. The sample was collected into the scintillation vials. 5 ml of Scintillation cocktail Econ2 (Fischer Scientific) was added to all scintillation vials. All vials were placed in a Beckmann LS6500 Beta liquid scintillation counter and counted for 10 min. Experiments were performed 1–3 times with 1–3 replicate determinations as indicated in Figure legends.

Once the less complete inhibitors of Thr uptake were identified from the more complete inhibitor series above, Leu and Pro, were used in the same experimental procedure using 0.5, 1.0, 5.0, 10.0, 20.0 mM of Leu and Pro, respectively, and 20 mM non-radioactive Thr in the NaCl uptake buffer. Our control was 50 uM ^3^H Thr uptake solution with no amino acid inhibitor.

Once the complete inhibitors of Thr uptake were identified from the complete inhibitor series, we conducted the same experimental procedure using 500 uM of only those complete inhibitors (Ser, Ala, Thr, Cys, Asn, 3-HNV, Met, 4-OH-Pro) in both the NaCl uptake buffer and no-Na^+^ uptake buffer, Choline Chloride. Our control was 50 uM ^3^H Thr uptake solution with no amino acid inhibitor.

### RT-PCR

For RNA isolation, approximately 5 × 10^6^ cells were spun down to a pellet at 300 × g for 5 min. 350 uL of Buffer RLT was added to lyse the pelleted cells. Total RNA was then isolated from the cells using the RNeasy Mini RNA Isolation Kit using the manufacturer protocol (Qiagen) (NOTE: The QIAshredder spin column protocol option was used). The RNA was quantified by measuring the absorbance at 260 nm. Typical total RNA yield was ~2300 ng/ul. The technique for RT-PCR was performed using 2 ug total RNA from the mES cells and the QIAGEN OneStep RT-PCR kit protocol that was recommended by the manufacturer. 0.6 uM PrimeTime qPCR Primers were used (Integrated DNA Technologies). The Slc1a4 and Slc1a5 qPCR primers amplify a ~150 bp PCR product. The sequence of the primers for Slc1a4 (ASCT1; Marin et al., 2000) are as follows: forward primer:

5′-TCATCCCTTCCACATCTGTTAC-3′; reverse primer:

5′- CCTGTTCCCTTCCAATCTTGT-3′. The sequence of the primers for Slc1a5 (ASCT2; Marin et al., 2000) are as follows: forward primer:

5′- CCATTCTTCTCCTCTACACACTTC-3′; reverse primer:

5′- CCTCTCATCTACTTCCTCTTCAC-3′. The no-template sample consisted of molecular biology grade water instead of RNA. The PrimeTime qPCR Primer stock solution was made to a concentration of 10 × according to the manufacturer's protocol (Integrated DNA Technologies). The RT-PCR conditions used were 30 min at 50°C, 15 min at 95°C, 1 min at 94°C, 1 min at 58°C, 1 min at 72°C, followed by 40 cycles of 1 min at 94°C, and then 10 min at 72°C.

### Gel electrophoresis

The gel was loaded and samples were electrophoresed at 100 V for 45 min in 1 × TAE buffer with ethidium bromide (0.5 ug/ml). The DNA was visualized using a UV transilluminator. Samples of cDNA were sent to Molecular Cloning Laboratories (San Francisco, CA) for sequencing. The sequences of the cDNAs corresponded to the sequences anticipated for those segments of ASCT1 and ASCT2 mRNA. The Slc1a4 forward and reverse primers (ASCT1) had a 98% match. The Slc1a5 forward and reverse primers (ASCT2) had a 99% match (Molecular Cloning Laboratories).

## Results

### Threonine has a Na^+^-dependent and Na^+^-independent component of transport

**Figure 1:**
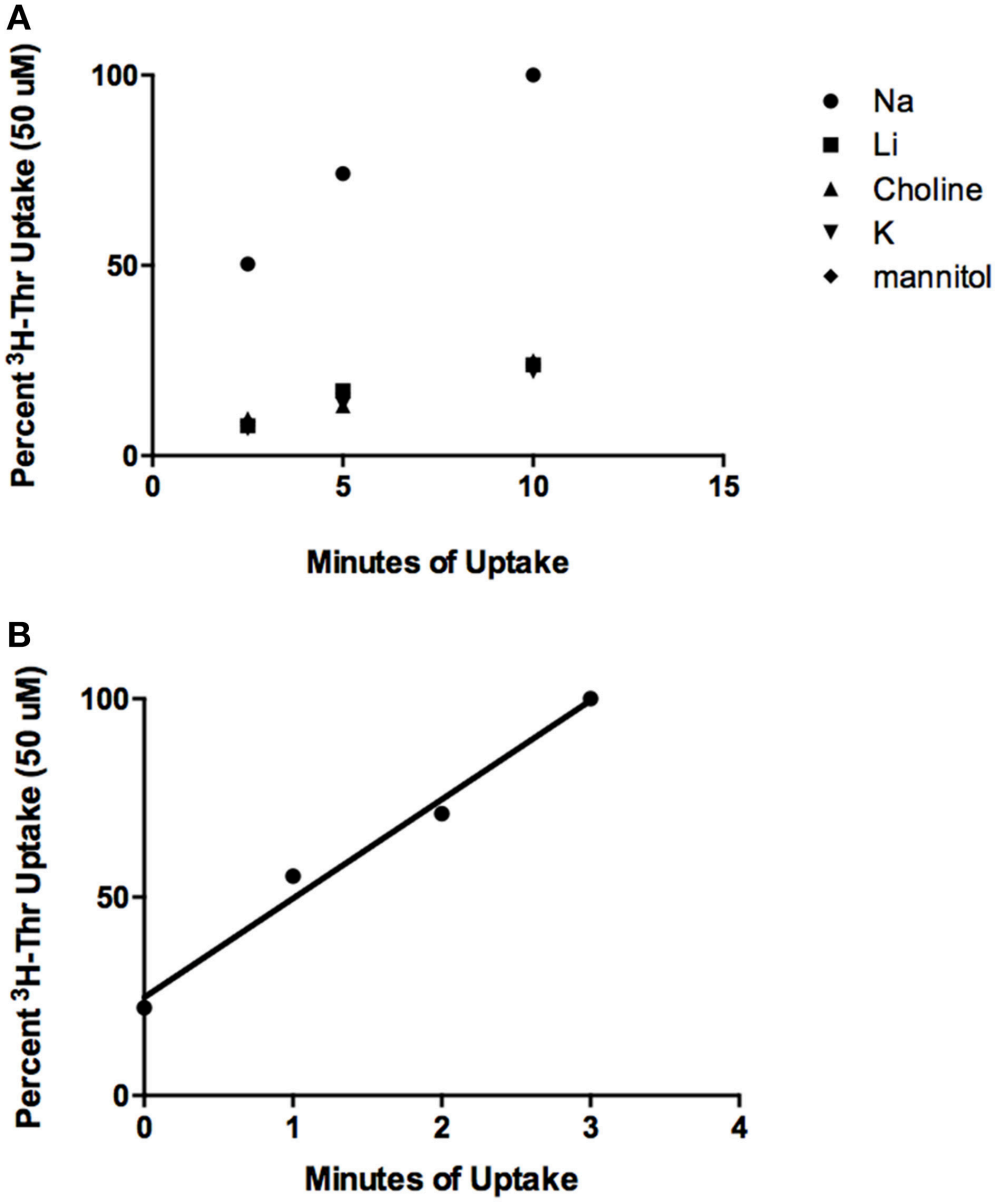
**Time course of 50 uM [^3^H]-Thr uptake in the presence of 140 mM NaCl, LiCl, Choline Cl, or KCl, or 280 mM Mannitol**. **(A)** Uptake was measured at 2.5, 5, and 10 min and increased significantly with time (*p* < 0.0001) (*n* = 3; 1 replicate experiment for each time point). **(B)** Time course of 50 uM [^3^H]-Thr uptake in the presence of 140 mM NaCl at 0, 1, 2, and 3 min. Uptake increased significantly with time (*r* = 0.99, *p* = 0.01, 1 replicate experiment for each time point).

From the time course studies, Thr uptake was much more rapid in the presence of Na^+^ than in its absence (Figure 1A, *p* < 0.003, Effect Size = 0.96). In both instances, Thr uptake increased nearly linearly with time for at least 10 min (*p* < 0.0001 for uptake in the absence of Na^+^ shown in the figure and in the presence of Na^+^ in a separate series of experiments with three determinations at each time point). The correlation coefficients for the increases of Thr uptake with time were: *r*_Na+_ = 0.97, *r*_no Na+_ = 0.96. The correlation coefficient for the increase in Thr uptake over 3 min was *r* = 0.99 (Figure 1B). Some binding of Thr to the cells also likely occurred since [^3^H]-Thr was associated with mES cells even after their exposure to [^3^H]-Thr for only a few seconds, as indicated at the 0 time point in Figure 1B.

### Threonine transport is inhibited to varying degrees by different amino acids in the presence or absence of Na^+^

**Figure 2:**
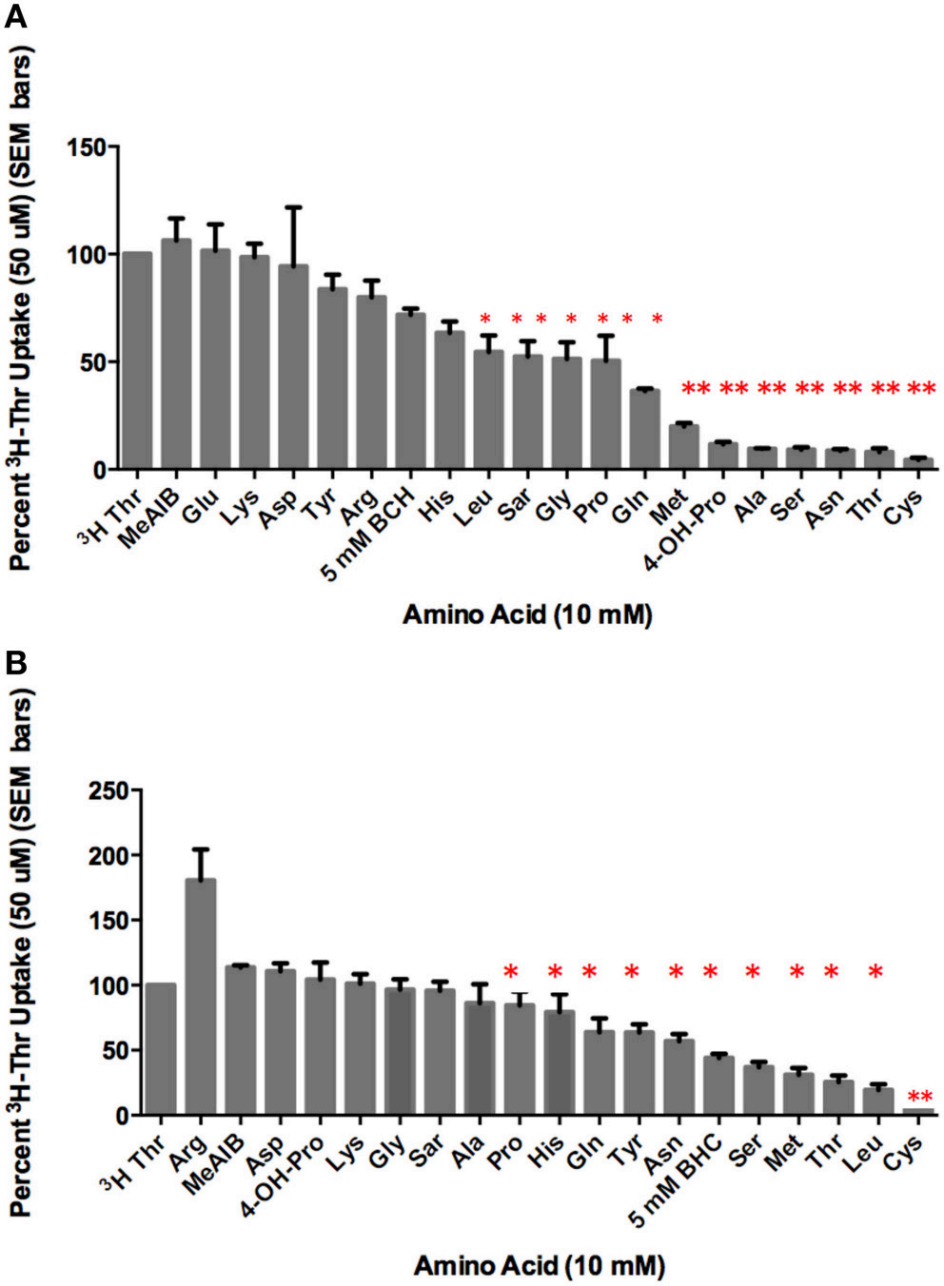
**Percent uptake of 50 uM [^3^H]-Thr in the presence of 140 mM NaCl and 10 mM of the amino acid indicated**. **(A)** A 1-sample *t*-test determined the more complete inhibitors (^**^p < 0.0001) and other statistically significant inhibitors (^*^p < 0.05) (*n* = 6 for each column; 3 determinations obtained in each of 2 independent experiments). **(B)** Percent uptake of 50 uM [^3^H]-Thr in the presence of 280 mM Mannitol and 10 mM of the amino acid indicated. A 1-sample *t*-test determined the more complete inhibitor (^**^p < 0.0001) and other statistically significant inhibitors (^*^p < 0.05) (*n* = 6 for each column; 3 determinations obtained in each of 2 independent experiments).

In the presence of Na^+^, [^3^H]-Thr transport was significantly inhibited (*p* < 0.05) by Ser, Gly, Leu, Pro, Sar, Cys Asn, Ala, Met, His, Gln, BCH, 4-OH-Pro, and non-radioactive Thr itself (Figure 2A). Of these, the more complete inhibitors were Ser, Thr, Cys, Asn, Ala, Met, and 4-OH-Pro (*p* < 0.0001; Figure 2A). Less complete inhibitors were Gln, Gly, Leu, Pro, His, Sar, and BCH. Arg, Tyr, Glu, Lys, meAIB, and Asp did not inhibit [^3^H]-Thr uptake to a statistically significant extent (Figure 2A). See Table S1 in the supplemental materials for a full list of the amino acids and their abbreviations.

In the absence of Na^+^, [^3^H]-Thr transport was significantly inhibited (*p* < 0.05) by Ser, Leu, MeAIB, Thr, Cys, Asn, Met, Gln, Tyr, and BCH (Figure 2B). Notably, Leu, Tyr, and BCH were better inhibitors in the absence than in the presence of Na^+^, while Asn and Ala were better inhibitors in the presence of Na^+^ (Figure 2A vs. Figure 2B; Table 1). Cys was the strongest inhibitor in both conditions (*p* < 0.0001) (Figures 2A,B). [^3^H]-Thr transport was not significantly inhibited by Glu, Lys, Gly, MeAIB, Pro, Asp, Sar, Ala, His, Arg, or 4-OH-Pro in the absence of Na^+^.

**Table 1:** **Mean % [^3^H]-Thr Uptake in 140 mM NaCl vs. 280 mM Mannitol and the amino acid indicated (from Figures 2A,B)**.

**Amino acid**	**NaCl uptake buffer (%)**	**Mannitol uptake buffer (%)**
Ser	9.12	37.05
Thr	8.00	25.53
Asn	8.68	57.05
Met	19.90	31.10
Gln	36.44	63.90
Leu	54.53	19.48
BCH	71.78	44.13
Tyr	83.64	63.65
Ala	9.56	86.20
Cys	4.40	14.78

### Establishing weaker vs. partial inhibition by Leu and Pro

**Figure 3:**
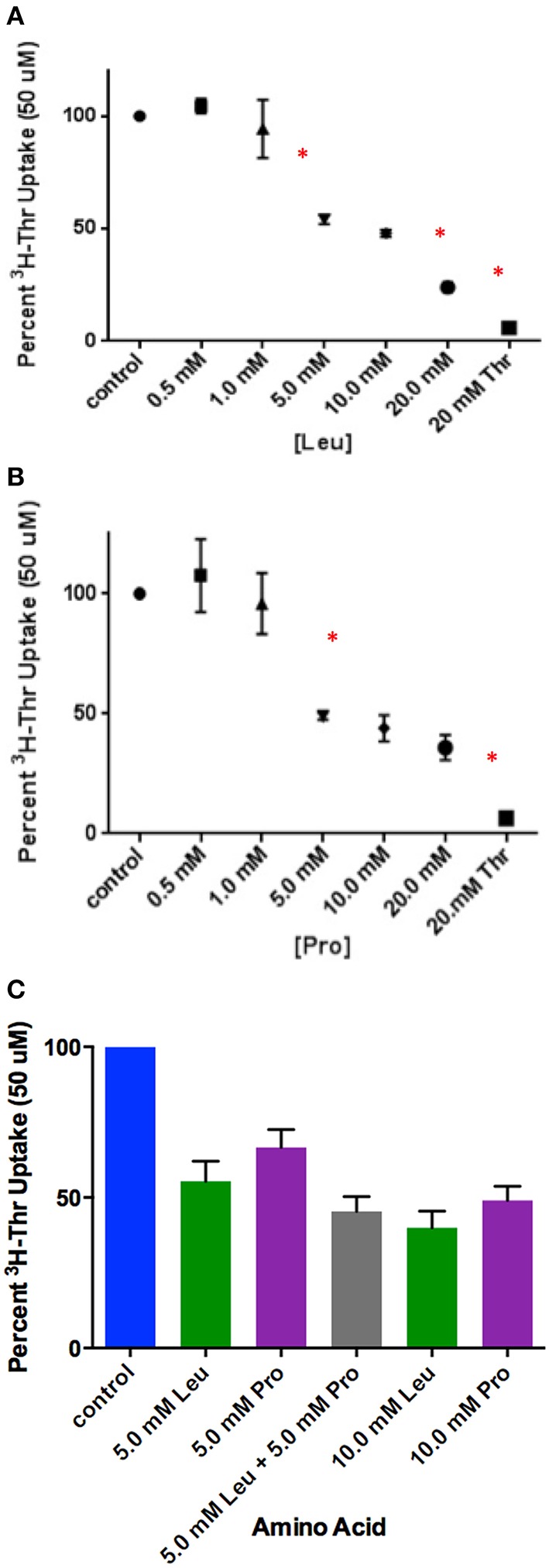
**Percent uptake of 50 uM [^3^H]-Thr in the presence of 140 mM NaCl and increasing concentrations of (A) Leu (*n* = 6 for each point; 3 determinations obtained in each of 2 independent experiments) or (B) Pro (*n* = 6 for each point; 3 determinations obtained in each of 2 independent experiments)**. Thr uptake was also measured in the presence of 20 mM non-radioactive Thr. A one-way ANOVA indicated statistically significant differences in [^3^H]-Thr uptake as the concentration of Leu and Pro increased. An asterisk (^*^) signifies significantly more inhibition by a treatment than the one that precedes it (*p* < 0.05). **(C)** Percent uptake of 50 uM [^3^H]-Thr in the presence of 140 mM NaCl and Leu, Pro, or Leu and Pro combined (*n* = 6 for each point; 3 determinations obtained in each of 2 independent experiments). A one-way ANOVA indicated no statistically significant differences (*p* > 0.05) in [^3^H]-Thr uptake except between 5 mM Pro and 10 mM Leu.

Of the incomplete inhibitors of Thr uptake in the presence of Na^+^ (Figure 1A), Leu and Pro were found to be weak rather than partial inhibitors. Thr uptake decreased as the Leu or Pro concentration increased to 20 mM (Figures 3A,B) indicating weak inhibition. Because Leu and Pro were weak inhibitors, they probably inhibited the same components of Thr transport. Furthermore, when Leu and Pro combined and examined as inhibitors of Thr uptake, Leu and Pro did not inhibit Thr transport more than Leu or Pro alone (Figure 3C). This confirmed that they were likely inhibiting the same Thr transporters.

### Determining relative transporter affinities for the stronger Thr inhibitors

**Figure 4:**
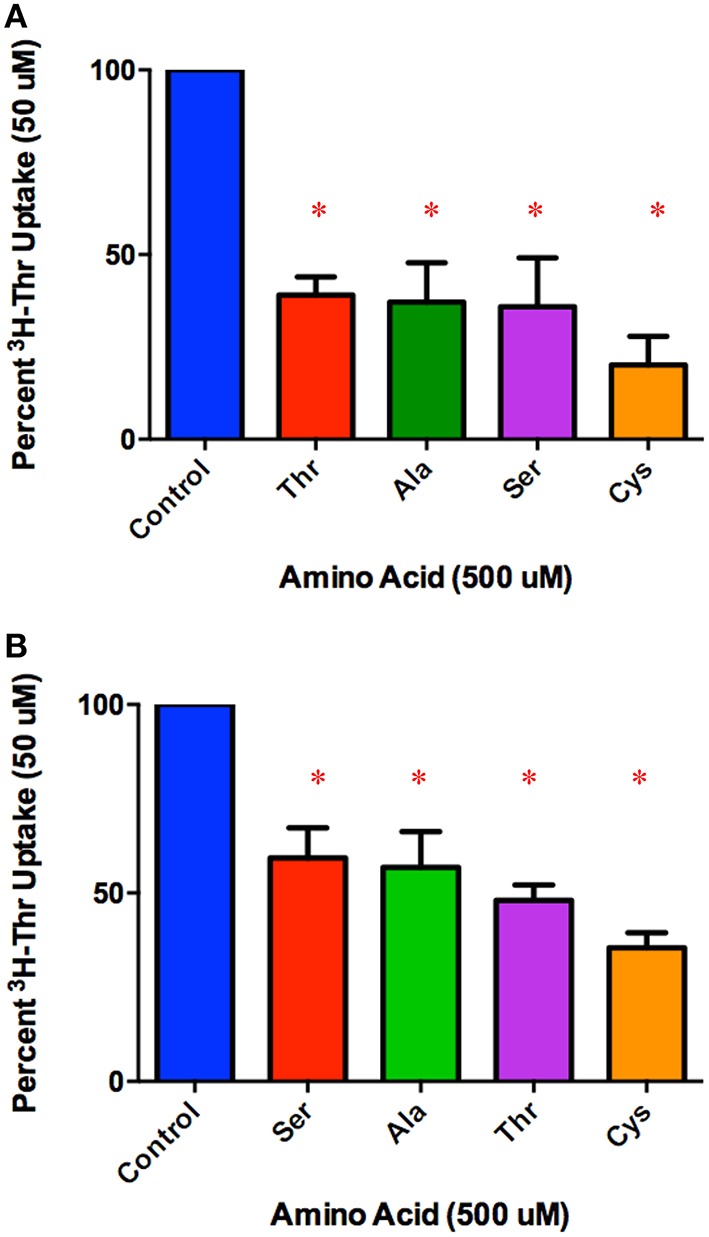
**Percent Na^+^-dependent uptake of 50 uM [^3^H]-Thr remaining in the presence of the indicated amino acid (500 uM) (A) after subtraction of uptake in 140 mM Choline Cl**. A 1-sample *t*-test confirmed that all amino acids tested were s significant inhibitors (^*^p < 0.05). A one-way ANOVA indicated no statistically significant difference in [^3^H]-Thr uptake in the presence of the different amino acids tested (*p* > 0.05) (*n* = 4 for each column; 2 determinations obtained in each of 2 independent experiments). **(B)** Percent uptake of [^3^H]-Thr remaining in the presence of the indicated amino acid (500 uM) but without subtraction of Na^+^-independent transport. A 1-sample *t*-test confirmed that all amino acids were statistically significant inhibitors (^*^p < 0.05). A one-way ANOVA indicated no statistically significant difference in [^3^H]-Thr uptake in the presence of the different amino acids tested (*p* > 0.05) (*n* = 4 for each column; 3 determinations obtained in each of 2 independent experiments).

Amino acids that inhibited 50 uM Thr uptake most completely were identified in Figure 2. Of these, the inhibition by Ser, Ala, non-radiolabeled Thr and Cys were compared at the lower concentration of 500 uM (Figure 4A). After subtracting Na^+^-independent Thr uptake, 500 uM Cys was the strongest inhibitor tested, with a mean proportion of Thr uptake remaining of only 20% (Figure 4A). However, no statistically significant differences in inhibition were seen amongst the 500 uM Ser, Ala, Thr, and Cys (*p* > 0.05). Even when the Na^+^-independent component of transport was not subtracted, the pattern of inhibition was the same (Figure 4A vs. Figure 4B).

**Figure 5:**
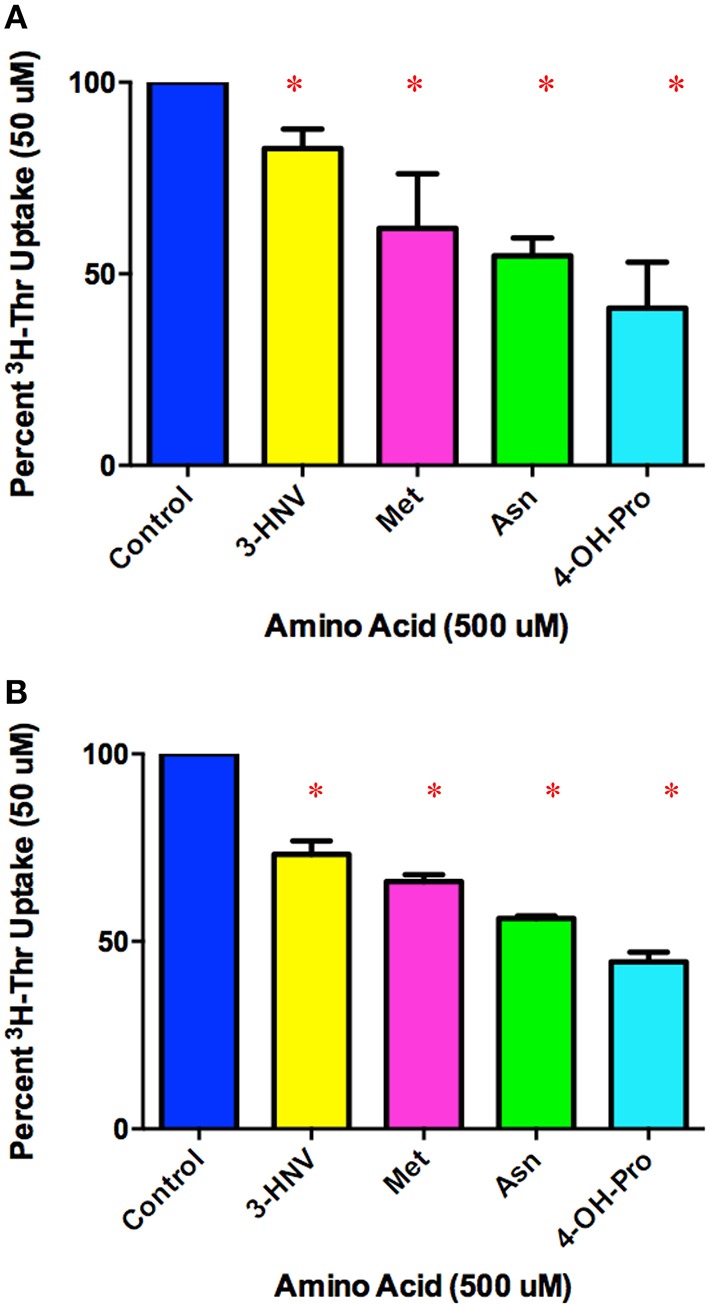
**Percent Na^+^-dependent uptake of 50 uM [^3^H]-Thr remaining in the presence of the indicated amino acid (500 uM) (A) after subtraction of uptake in 140 mM Choline Cl**. A 1-sample *t*-test confirmed that all amino acids were statistically significant inhibitors (^*^p < 0.05). A one-way ANOVA indicated no statistically significant difference in [^3^H]-Thr uptake in the presence of the different amino acids tested (*p* > 0.05) (*n* = 4 for each column; 2 determinations obtained in each of 2 independent experiments). **(B)** Percent uptake of [^3^H]-Thr remaining in the presence of the indicated amino acid (500 uM) but without subtraction of Na^+^-independent transport. A 1-sample *t*-test confirmed that all amino acids were statistically significant inhibitors (^*^p < 0.05). A one-way ANOVA indicated 4-OH-Pro inhibited [^3^H]-Thr uptake significantly more than the other amino acids tested (*p* < 0.05). (*n* = 4 for each column; 2 determinations obtained in each of 2 independent experiments).

**Figure 6:**
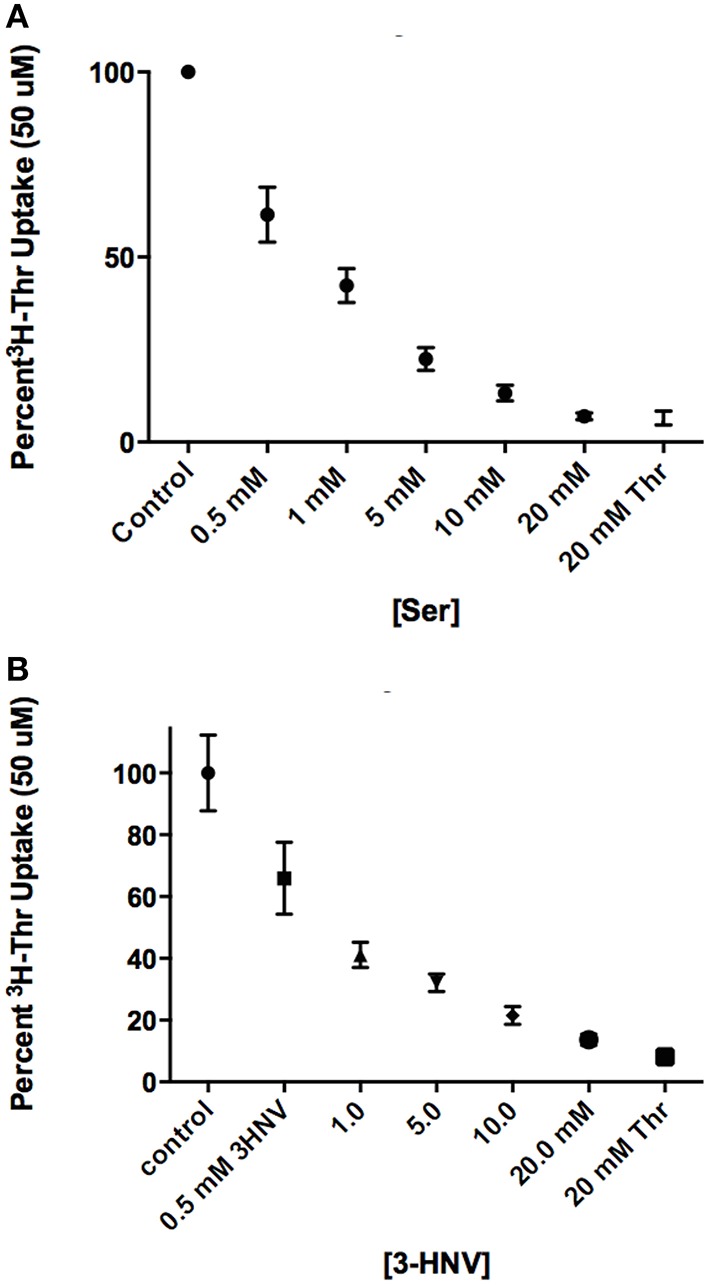
**Percent uptake of 50 uM [^3^H]-Thr in the presence of 140 mM NaCl and increasing concentrations of (A) Ser or (B) 3-HNV (*n* = 6 for each point; 3 determinations obtained in each of 2 independent experiments)**. Thr uptake was also measured in the presence of 20 mM non-radiolabeled Thr. A 2-sample *t*-test indicated that [^3^H]-Thr uptake in 20 mM Ser was not significantly different from [^3^H]-Thr uptake in 20 mM non-radiolabeled Thr (*p* > 0.05), whereas [^3^H]- Thr uptake in 20 mM 3-HNV was significantly higher than [^3^H]- Thr uptake in 20 mM non-radiolabeled Thr (*p* < 0.05). A one-way ANOVA also indicated that 50 uM [^3^H]-Thr uptake significantly decreased with increasing concentration of Ser or 3-HNV (*p* < 0.05).

In comparing a second set of more complete inhibitors identified in Figure 2A (Asn, Met, 3-HNV, and 4-OH-Pro), 500 uM 4-OH-Pro was the strongest inhibitor tested, with a mean proportion of Thr uptake remaining of 41% after subtracting Na+-independent Thr transport (Figure 5A). However, no statistically significant differences in inhibition were found amongst 500 uM Asn, Met, 3-HNV, and 4-OH-Pro (*p* > 0.05). The pattern of inhibition was the same even when the Na^+^-independent component of transport was not subtracted (Figure 5A vs. Figure 5B) although inhibition by 4-OH-Pro was then greater than the other inhibitors tested (*p* < 0.05) (Figure 5B). Additionally, although a good inhibitor, 3-HNV was not one of the strongest inhibitors (Figures 5A,B). For example, 20 mM Ser inhibited [^3^H]-Thr uptake almost as much as 20 mM non-radiolabeled Thr (Figure 6A), whereas 20 mM 3-HNV inhibited [^3^H]-Thr uptake considerably less completely than did 20 mM non-radiolabeled Thr (*p* < 0.05; Figure 6B).

### Determining mRNA encoding ASCT1/2 via RT-PCR

**Figure 7:**
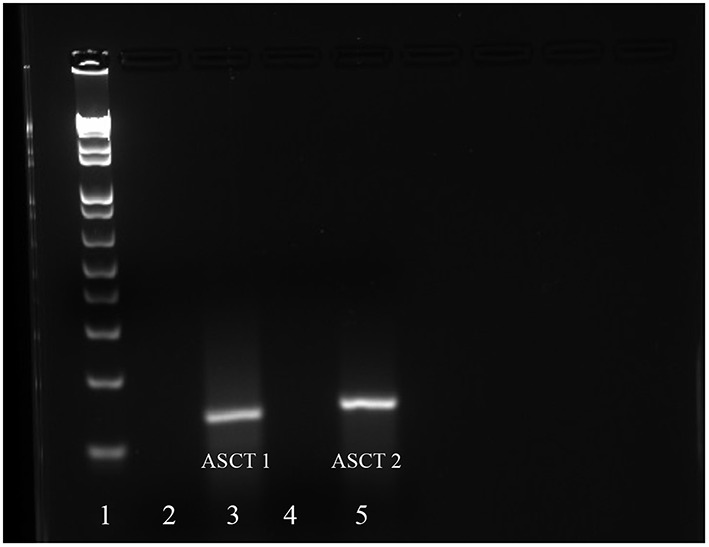
**mRNA expression of ASCT1 and ASCT2 in mES cells as determined by RT-RCR**. Lane 1: DNA ladder; Lane 2: ASCT1 primers and no RNA template control; Lane 3: ASCT1 primers and template RNA; Lane 4: ASCT2 primers and no template RNA control; Lane 5: ASCT2 primers and template RNA.

Given the findings from our transport studies, it was likely that the Na^+^-dependent transporters, ASCT1 and/or ASCT2, were responsible for most of the Na^+^-dependent transport of Thr in mES cells. Upon conducting RT-PCR and gel electrophoresis, we detected cDNA bands of the size anticipated for both ASCT1 and ASCT2 mRNA (Figure 7). The sequences of the cDNAs corresponded exactly to the sequences anticipated for those segments of ASCT1 and ASCT2 mRNA (Molecular Cloning Laboratories) (Data not shown).

## Discussion

### mES cells express both Na^+^-dependent and Na^+^-independent components of thr transport

As depicted in the time course studies, Na^+^-dependent Thr uptake is considerably more rapid than Na^+^-independent uptake in mES cells (Figure 1A). Nevertheless, Na^+^-independent Thr transport is substrate saturable as there was non-radiolabeled Thr inhibition of [^3^H]- Thr transport in the absence of Na^+^ (Figure 2B). Hence, mES cells express both Na^+^-dependent and Na^+^-independent components of Thr transport.

### Amino acid inhibition of Na^+^-dependent Thr transport in mES cells resembles such inhibition of amino acid transport system ASC in other cells

Thr, Ser, Cys, 4-OH-Pro, Asn, and Ala were relatively strong inhibitors of the Na^+^-dependent [^3^H]-Thr transport in mES cells (Figures 2A, 4B, 5B). These amino acids are also relatively strong inhibitors for the ASCT1 and ASCT2 transports in other cell types (Utsunomiya-Tate et al., 1996; Yamamoto et al., 2003, 2004). Specifically, both the Na^+^-dependent Thr transporter(s) in mES cells and the ASC system transporters have a strong preference for neutral amino acids without branched or bulky side chains, with the exception that they tolerate a hydroxyl or sulfhydryl group at C3 or C4 of the amino acid (Utsunomiya-Tate et al., 1996; Pinilla-Tenas et al., 2003). Thr, Ser, 3-HNV, Cys, and 4-OH-Pro, share these characteristics. The hydroxylation of Pro at C4 greatly increases its affinity for both the ASC system transporters (Utsunomiya-Tate et al., 1996; Pinilla-Tenas et al., 2003) and the Na^+^-dependent component of transport in mES cells. For example, 0.5 mM Pro does not inhibit Thr uptake (Figure 3B) whereas this concentration of 4-OH-Pro does inhibit Thr transport (Figure 5B). Asn is another relatively strong inhibitor of Na^+^-dependent Thr uptake in both mES cells (Figures 2A, 5B) and the ASC system in other cells (Utsunomiya-Tate et al., 1996). While Asn does not have a hydroxyl or sulfhydryl group, it does have polar groups on C4, similar to 4-OH-Pro. This could explain its relatively strong inhibitor properties.

Ala, Met, and Gln were also good inhibitors of Na^+^-dependent Thr uptake (Figures 2A, 4B, 5B). Ala inhibition could be attributed to its small neutral structure, and both the ASCT1 and ASCT2 transporters are selective for small amino acids (Utsunomiya-Tate et al., 1996). As for Met and Gln inhibition in mES cells, ASCT2 (but not ASCT1) has been shown to interact strongly with both of these amino acids in other cell types (Utsunomiya-Tate et al., 1996; Bröer et al., 1999). Thus, Ala might inhibit both Na^+^-dependent ASCT1 and ASCT2 transporters in mES cells, while Met and Gln might inhibit mainly ASCT2 in these cells. This could account for the greater inhibition of Thr uptake seen by 10 mM Ala compared to 10 mM Met and Gln (Figure 2A).

Lastly, 10 mM Gly, Leu, Pro, and His were incomplete inhibitors of Thr uptake in mES cells (Figure 2A), and ASCT2 interacts weakly with these amino acids (Utsunomiya-Tate et al., 1996). Specifically, Thr uptake decreased as the Leu or Pro concentration increased to 20 mM (Figures 3A,B), indicating that Leu and Pro were probably inhibiting the same components of Thr transport. Additionally, since Leu and Pro did not inhibit Thr transport more when combined than when studied separately (Figure 3C), they were probably inhibiting the same Na^+^-dependent transporters.

### ASCT1 and ASCT2 mRNA are present in mES cells

These findings for Thr transport in mES cells support the theory that the cells express one or both of the system ASC transporters, ASCT1 and ASCT2. RT-PCR and sequencing of the cDNA products confirmed expression of mRNA encoding both ASCT1 and ASCT2 in mES cells (Figure 7). Thus, it is likely that ASCT1 and ASCT2 are responsible for Na^+^-dependent Thr transport in mES cells, and that there are at least two components of Na^+^-dependent transport in the cells.

### Amino acid inhibition of Na^+^-independent Thr transport in mES cells resembles such inhibition of amino acid transport system L in other cells

Ser, Leu, Cys, Asn, Met, Gln, Tyr, BCH, and non-radiolabeled Thr inhibited the Na^+^-independent component of [^3^H]-Thr transport in mES cells (Figure 2B). Such is also the case for System L transporters in other cell types (Segawa et al., 1999). Furthermore, both the Na^+^-independent Thr transporter(s) in mES cells (Figure 2B) and System L transporters in other cell types are inhibited strongly and selectively by the Leu analog, BCH (Segawa et al., 1999; Fukasawa et al., 2000; Fuchs and Bode, 2005). Finally, Tyr inhibited Thr transport only in the absence of Na^+^ (Figure 2B). LAT2, a system L transporter with broad substrate selectivity, also interacts with Tyr (Fukasawa et al., 2000; Fuchs and Bode, 2005). Thus, the Na^+^-independent Thr transporter(s) in mES cells is likely LAT2 or another system L transporter.

## Conclusion

This is the first study to characterize Thr transport by mES cells. Our findings support the conclusion that ASCT1 and ASCT2 are primarily responsible for the Na^+^-dependent component of Thr transport in these cells. Because these two transporters are so similar, however, studying them independently for Thr transport in mES cells is difficult. Both are likely present and both probably play roles in transporting Thr into the cells. While there is also a Na^+^-independent component of Thr transport in mES cells, it was less well characterized in these studies. According to its transport characteristics, however, it is probably a form of amino acid transport system L.

Future studies could explore the Na^+^-independent component of Thr transport in mES cells in more detail. Additionally, now that the transporters responsible for the majority of Thr transport into mES cells have been identified, future studies should explore the effect Thr transport inhibitors have on the growth of mES cells. If mES cell growth is arrested when Thr transport is inhibited by other amino acids such as Ser, Cys, or Ala, then this would support the conclusion that Thr transport mediates Thr's effects in controlling mES cell proliferation. In this regard, our preliminary studies show that both Ser and 3-HNV inhibit proliferation of mES cells, although Ser inhibition is not nearly as strong as that of 3-HNV (Rasmussen and Van Winkle, personal communication). In this regard, the stoichiometry of Na^+^/amino acid cotransport by system ASC varies from 4.5 to 0.22 depending on the structure of the amino acid (Van Winkle, 2001). Such variable interactions of amino acids with the ASC transport protein could also mean that they influence signaling initiated by the protein (Ryu and Han, 2011) in different ways or to varying extents. Moreover, it would be interesting in future studies to knockdown either or both ASCT1 or ASCT2 expression and measure the effects of such knockdowns on mES cell proliferation.

## Author contributions

TF is the primary author/researcher on this paper. She conducted all experiments and did all the writing for the project under the guidance of her primary investigator, LV. LV and TF worked together to develop the project, protocols for the experiments, and analysis of results.

## Funding

Midwestern University Department of Biomedical Sciences provided all funding for this project.

### Conflict of interest statement

The authors declare that the research was conducted in the absence of any commercial or financial relationships that could be construed as a potential conflict of interest.
