# Supplementary material for: At Least Three Transporters Likely Mediate Threonine Uptake Needed for Mouse Embryonic Stem Cell Proliferation
Source: Front Cell Dev Biol. 2016 Mar 15;4:17. doi: 10.3389/fcell.2016.00017 (PMC4791362; doi:10.3389/fcell.2016.00017)
Supplement: Supplementary file 1 [file Table1.docx]

**Supplemental Materials**

**Table 1S. Acronyms for various amino acids and amino acid analogues mentioned in paper.**

| **Acronym** | **Meaning** | **Amino acid/Analogue** |
| --- | --- | --- |
| 3-HNV | 3-hydroxynorvaline | Analogue |
| 4-OH-Pro | 4-hydroxy-proline | Analogue |
| Ala | Alanine | Amino acid |
| Arg | Arginine | Amino acid |
| Asn | Asparagine | Amino acid |
| Asp | Aspartate | Amino acid |
| BCH | *z*-amino*endo*bicyclo-[2,2,1]-heptane-2-carboxylic acid | Analogue |
| Cys | Cysteine | Amino acid |
| Gln | Glutamine | Amino acid |
| Glu | Glutamate | Amino acid |
| Gly | Glycine | Amino acid |
| His | Histidine | Amino acid |
| Leu | Leucine | Amino acid |
| Lys | Lysine | Amino acid |
| MeAIB | Methylaminoisobutyric acid | Analogue |
| Met | Methionine | Amino acid |
| Pro | Proline | Amino acid |
| Sar | Sarcosine | Analogue |
| Ser | Serine | Amino acid |
| Thr | Threonine | Amino acid |
| Tyr | Tyrosine | Amino acid |
